# Liquid Markers for Risk Stratification of Pulmonary Nodules, Ready for Prime Time? Yes!

**DOI:** 10.1016/j.chpulm.2024.100071

**Published:** 2024-06-11

**Authors:** Roger Y. Kim, Catherine R. Sears, Nicholas J. Pastis

**Affiliations:** Division of Pulmonary, Allergy and Critical Care (R. Y. K.), Department of Medicine, University of Pennsylvania, Philadelphia, PA; the Division of Pulmonary, Critical Care, Sleep and Occupational Medicine (C. R. S.), Department of Medicine, Indiana University School of Medicine, Indianapolis, IN; the Division of Pulmonary Medicine (C. R. S.), Richard L. Roudebush Veterans Affairs Medical Center, Indianapolis, IN; and the Division of Pulmonary, Critical Care and Sleep Medicine (N. J. P.), Department of Internal Medicine, The Ohio State University College of Medicine, Columbus, OH.

Indeterminate pulmonary nodules are commonly identified on lung imaging and represent a challenge for physicians, who estimate malignancy risk to decide how best to manage these lesions that could ultimately represent either lung cancers or benign processes.^1^ For those nodules at higher malignancy risk, a lung biopsy, surgical resection, or empirical treatment is recommended. For low-risk nodules, serial surveillance with CT imaging is indicated.^2^ However, even validated malignancy risk estimations are not universally accurate and carry the inherent risks of performing unnecessary invasive procedures for benign nodules or of delayed diagnosis and possible upstaging of lung cancers.^3^ In fact, physician assessment of risk has been shown to outperform traditional clinical risk models; however, physicians often ignore guideline-based recommendations for the next diagnostic test in the workup of a pulmonary nodule.^4^ The promise of noninvasive biomarkers is their potential to enhance clinical decision-making in these challenging circumstances. To ground any discussion regarding the prime time status of liquid biomarkers for risk stratification of pulmonary nodules, we must first agree on the definition of prime time. We argue that because there is clearly a definitive lack of clinical utility evidence in support of using liquid biomarkers for risk stratification of pulmonary nodules in routine clinical practice, prime time for this pro-con debate should be defined as follows: ready for clinical implementation based on efficacy extrapolated from multiple rigorous clinical validation and registry studies. The official American Thoracic Society^5^ policy statement on lung cancer biomarkers, published in 2017, directly addressed the challenges of a criterion standard clinical utility study. The authors concluded that “large, well-designed and conducted studies, capable of determining the impact of testing on clinical decisions and outcomes, are required to confirm the clinical utility of a molecular biomarker.”^5^ We argue that although there are currently no published prospective randomized controlled trials assessing the clinical utility of pulmonary nodule biomarkers that fit these criteria, several biomarkers have demonstrated promise through consistent results across multiple clinical validation studies and prospectively collected data from registries, and some are already commercially available.

Numerous diagnostic biomarkers have been developed over the past two decades to assist in earlier diagnosis of lung cancer. These measure proteins, microRNA, circulating tumor cells, circulating tumor DNA, and methylated DNA, among others.^6^ The focus of this point/counterpoint is on liquid biomarkers, but it warrants mentioning that these concepts also largely pertain to commercially available biomarkers that measure epithelial gene expression profiling. We will focus our discussion predominantly on peripheral blood-based biomarkers, which can be categorized as measuring either tumor-specific antigens or tumor-associated antibodies. These biomarkers have been assessed either alone or in combinatory panels with or without the incorporation of clinical risk factors. Commercially available examples include Nodify XL2 (Biodesix), which incorporates five clinical risk factors (age, smoking status, nodule diameter, nodule border characteristics, and nodule location) and the use of multiple reaction monitoring mass spectroscopy to identify two proteins,^7^ and Nodify CDT (Biodesix), which uses a tumor-associated antibody panel.^8^ The Pulmonary Nodule Proteomic Classifier (PANOPTIC) trial was a multicenter prospective observational study that retrospectively evaluated the performance of the Nodify XL2. Among the 178 individuals with pulmonary nodules determined by a pulmonologist or thoracic surgeon to have a probability of cancer ≤ 50% (cancer prevalence of 16%), Nodify XL2 classified 66 (37%) as likely benign, of which 65 were determine to have a benign diagnosis (negative predictive value of 98%).^7^ Thus, this test may be useful among individuals with nodules with low-to-intermediate pretest probability of malignancy to assist in excluding malignancy. On the other hand, the Nodify CDT test may be useful in ruling in malignancy among individuals with higher pretest probabilities of malignancy. In a separate study including 296 individuals with a cancer prevalence of 25%, the addition of the Nodify CDT test to the Mayo Clinic clinical risk model increased specificity at increasing malignancy risk thresholds.^8^ These published studies on liquid biomarkers represent a key first step to develop and validate promising risk prediction tools, as suggested by the American Thoracic Society policy statement.^5^ In the meantime, well-designed prospective randomized controlled trials must be undertaken to confirm the clinical utility of liquid biomarkers. Because clinical validation studies are associated with very defined criteria for interpretation and physician utilization, these studies should particularly determine the following: (1) if outcomes support those determined by extrapolation from prior studies and (2) if there is increased benefit or reduction in harm compared with the current standard of care. This is particularly important because biomarker utilization outside of the studied patient populations, and misinterpretation and misuse of biomarker results, may result in reduced accuracy or even harm to patients. There is currently an enrolling clinical trial sponsored by Biodesix^9^ that seeks to randomize individuals to one of the two following pulmonary nodule risk stratification strategies: (1) open-label use of the Nodify XL2 test or (2) usual care blinded to the results of the Nodify XL2 test. Importantly, it will provide a platform by which important elements to ensure accurate use and interpretation (eg, test ordering, clear result sharing, interpretation) can be optimized for best clinical practice. The launching of this and other similar studies represents strong evidence that liquid biomarkers are ready for clinical implementation and moving ever closer to routine clinical use.

From a pragmatic standpoint, physician skill and comfort in managing intermediate-risk pulmonary nodules varies,^10^ physicians do not always follow guidelines,^4^ and there is not always a standard of care for each situation.^2^ Therefore, there is need for an additional validated datapoint to guide decision-making that liquid biomarkers can fill. Even in patients with significant risk factors for lung cancer as in the US National Lung Screening Trial, there was a nearly 30% false-positive rate with CT screening, frequently due to noncalcified granulomas which also tend to be falsely positive on PET imaging.^11,12^ Nevertheless, a solitary pulmonary nodule is most commonly considered indeterminate on imaging, unless it demonstrates an overt radiologic feature favoring benignity.^13^ A biomarker provides the additional datapoint to classify a nodule as very low risk for malignancy when other false-positive or nondiagnostic tests may lead physicians down the path of unnecessary invasive procedures or even surgery. The reliability of a negative biopsy for suspected lung cancer (transthoracic or bronchoscopic) has long been called into question, and this conundrum is now heightened with increasing use of novel peripheral bronchoscopy platforms, which have expanded the access to nodules for sampling but may be less reliable as rule out tests.^14^ An emerging use for biomarkers is in the setting of a negative bronchoscopy among patients with an intermediate risk of lung cancer (ie, < 66%). Although not a liquid biomarker, an RNA biomarker from bronchial brushings has demonstrated that the posttest probability of malignancy was decreased to < 10% when bronchoscopic findings and the classifier score were negative.^15^ This classifier may help avoid invasive procedures 50% of the time in nodules with low and intermediate pretest risk of malignancy.^16^ In addition, recommendations for surgical resection were higher with a positive compared with negative biomarker result, indicating that fewer patients requiring surgical referral were inappropriately observed with further surveillance imaging.^17^ This is relevant because it has been shown that physicians may inappropriately observe high-risk nodules when they should refer to surgery instead.^4,18^

In conclusion, we should consider the core tenet of what constitutes the ideal biomarker for pulmonary nodule risk stratification: a tool that influences clinical decision-making to improve patient care by providing a rationale for noninvasive surveillance of benign nodules or for more aggressive surgical referral when necessary.^5^ We argue that liquid biomarkers have demonstrated efficacy extrapolated from multiple rigorous clinical validation and registry studies and are capable of assisting physicians in classifying nodules as very low or high risk without substantially increasing the number of invasive diagnostic procedures performed for benign nodules or delaying therapeutic procedures for malignant nodules. Thus, they are ready for prime time clinical implementation.

## ABBREVIATIONS:

AEGIS: Airway Epithelial Gene Expression in the Diagnosis of Lung Cancer
ATS: American Thoracic Society
pCA: probability of malignancy
